# Characterization of the Common Genetic Variation in the Spanish Population of Navarre

**DOI:** 10.3390/genes15050585

**Published:** 2024-05-04

**Authors:** Alberto Maillo, Estefania Huergo, María Apellániz-Ruiz, Edurne Urrutia-Lafuente, María Miranda, Josefa Salgado, Sara Pasalodos-Sanchez, Luna Delgado-Mora, Óscar Teijido, Ibai Goicoechea, Rosario Carmona, Javier Perez-Florido, Virginia Aquino, Daniel Lopez-Lopez, María Peña-Chilet, Sergi Beltran, Joaquín Dopazo, Iñigo Lasa, Juan José Beloqui, NAGEN-Scheme, Ángel Alonso, David Gomez-Cabrero

**Affiliations:** 1 Translational Bioinformatics Unit, Navarrabiomed, Hospital Universitario de Navarra (HUN), Universidad Pública de Navarra (UPNA), IdiSNA, 31008 Pamplona, Spain; alberto.ruizdeinfante@kaust.edu.sa (A.M.);; 2 Biological and Environmental Science and Engineering Division, King Abdullah University of Science and Technology (KAUST), Thuwal 23955-6900, Saudi Arabia; 3 Genomics Medicine Unit, Navarrabiomed, Hospital Universitario de Navarra (HUN), Universidad Pública de Navarra (UPNA), IdiSNA, 31008 Pamplona, Spain; 4 Servicio de Genética Médica, Hospital Universitario de Navarra (HUN), 31008 Pamplona, Spain; 5 Dp. Bioquímica y Biología Molecular, Universidad Pública de Navarra (UPNA), 31006 Pamplona, Spain; 6 Instituto de Genética Médica y Molecular (INGEMM), Hospital Universitario La Paz, 28046 Madrid, Spain; 7 Department of Personalized Medicine, NASERTIC, Government of Navarra, 31011 Pamplona, Spain; 8 Computational Medicine Platform, Andalusian Public Foundation Progress and Health-FPS, 41013 Sevilla, Spain; 9 Institute of Biomedicine of Seville, IBiS, University Hospital Virgen del Rocio/CSIC/University of Sevilla, 41013 Sevilla, Spain; 10 FPS/ELIXIR-ES, Fundación Progreso y Salud (FPS), CDCA, Hospital Virgen del Rocio, 41013 Sevilla, Spain; 11 Biomedical Research Networking Center in Rare Diseases (CIBERER), Health Institute Carlos III, 28029 Madrid, Spain; 12 Centro Nacional de Analisis Genomico (CNAG-CRG), Centre for Genomic Regulation (CRG), The Barcelona Institute of Science and Technology, 08028 Barcelona, Spain; 13 Universitat Pompeu Fabra (UPF), 08002 Barcelona, Spain; 14 Departament de Genètica, Microbiologia i Estadística, Facultat de Biologia, Universitat de Barcelona (UB), 08007 Barcelona, Spain; 15 Laboratory of Microbial Pathogenesis, Navarrabiomed, 31008 Pamplona, Spain

**Keywords:** personalized medicine, whole genome sequencing WGS, whole exome sequencing WES, single nucleotide variant SNV, population frequencies, genetic variability

## Abstract

Large-scale genomic studies have significantly increased our knowledge of genetic variability across populations. Regional genetic profiling is essential for distinguishing common benign variants from disease-causing ones. To this end, we conducted a comprehensive characterization of exonic variants in the population of Navarre (Spain), utilizing whole genome sequencing data from 358 unrelated individuals of Spanish origin. Our analysis revealed 61,410 biallelic single nucleotide variants (SNV) within the Navarrese cohort, with 35% classified as common (MAF > 1%). By comparing allele frequency data from 1000 Genome Project (excluding the Iberian cohort of Spain, IBS), Genome Aggregation Database, and a Spanish cohort (including IBS individuals and data from Medical Genome Project), we identified 1069 SNVs common in Navarre but rare (MAF ≤ 1%) in all other populations. We further corroborated this observation with a second regional cohort of 239 unrelated exomes, which confirmed 676 of the 1069 SNVs as common in Navarre. In conclusion, this study highlights the importance of population-specific characterization of genetic variation to improve allele frequency filtering in sequencing data analysis to identify disease-causing variants.

## 1. Introduction

In recent years, the use of NGS in patient healthcare has increased due to technological advances, cost reduction, and enhanced efficiency [1]. The advancement of NGS spans a spectrum of applications, encompassing whole exome/genome sequencing (WES/WGS). These technologies revealed a wealth of genetic variants, necessitating the implementation of filters to narrow down the list of candidate variants. In this regard, the availability of population-specific catalogues of common variants enables the identification of rare variants [2], such as the international initiatives 1000 Genome Project (1KGP) [3] and Genome Aggregation Database (gnomAD) [4]. Moreover, various countries like the UK [5], USA [6], and Japan [7] have established their databases. In Spain, for instance, the Medical Genome Project (MGP) compiles data from unrelated healthy individuals [8,9].

In Navarre, the region of north-eastern Spain populated by 650,000 people, the local Government supported the “NAGEN scheme” to integrate genomic data analysis into the regional public healthcare system. In recent times, NAGEN has generated numerous WES/WGS and associated phenoclinical profiles in seven projects, including *NAGEN1000*, focusing on rare diseases and *pharmaNAGEN* on pharmacogenomics in patients with inflammatory bowel diseases [10]. The NAGEN strategy’s success hinges on identifying population-specific common variants to establish a comprehensive Navarrese population frequency catalogue.

**Figure 1:**
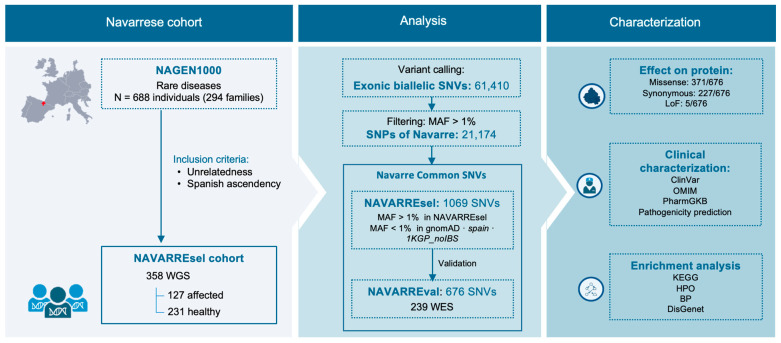
Workflow of this study. Abbreviations: *MGP*, Medical Genome Project; *1KGP*, 1000 Genomes Project; *1KGP_noIBS*, 1000 Genomes Project without Iberian population; *gnomAD,* Genome Aggregation Database; *MAF*, minor allele frequency; *SNV*, single nucleotide variant; *SNP*, single nucleotide polymorphism; *WGS*, whole genome sequencing; *WES*, whole exome sequencing; *LoF*, Loss-of-function; HPO, Human Phenotype Ontology; BP, biological process.

In this study (Figure 1), we aimed to identify and characterize common exonic variants specific to the Navarrese population. Firstly, we identified common single nucleotide variants (SNVs) in Navarre that are rare in other populations. Secondly, we validated the allele frequency of these variants in another Navarrese cohort with exome data. Finally, we annotated the resulting variants using genomic databases, and their clinical and pharmacological effects and pathogenicity were assessed. Additionally, we conducted functional enrichment analyses to provide further insights. The results will significantly contribute to advancing personalized medicine in Navarre.

## 2. Material and Methods

### 2.1. NAGEN1000 and pharmaNAGEN

In Navarre, the local Government supports the “NAGEN scheme”, integrating genomic data analysis into regional healthcare across seven projects. In the *NAGEN1000* project, 688 participants were recruited to uncover the underlying genetic causes of disease using WGS data. These individuals belonged to 294 families, predominantly trios, affected by rare disorders. Likewise, in the *pharmaNAGEN* project, 274 patients with Crohn’s disease or ulcerative colitis were recruited to analyse variants related to drug efficacy and toxicity on WES data [10]. All individuals recruited for these projects are part of the current population of Navarre and reside in the region.

### 2.2. Whole Genome Sequencing and Data Analysis

High-quality DNA samples from peripheral blood were used in the *NAGEN1000* project to construct short-insert paired-end libraries with an average insert size of 400 bp. DNA fragmentation was performed with Covaris S2, and capillary electrophoresis was performed with a Bioanalyzer 2100 (Agilent, Santa Clara, CA, USA). Libraries were sequenced on a NovaSeq 6000 (Illumina, San Diego, CA, USA) with a read length of 2 × 150 bp. A 30X coverage per sample was targeted. After quality control assessment using FASTQC (https://www.bioinformatics.babraham.ac.uk/projects/fastqc/, accessed on 10 October 2017), the sequenced data were aligned to the hs37d5 version of human genome reference GRCh37/hg19 using GEM3 [11]. Optical and duplicated reads were flagged with Picard MarkDuplicates (https://broadinstitute.github.io/picard/, accessed on 10 October 2017). Following the established Genome Analysis Toolkit (GATK) best practices pipeline (v3.8) [12], indel realignments and recalibration were applied to the previous BAM files. Variant calling on each sample’s BAM file was performed using HaplotypeCaller, with default parameters, resulting in a gVCF file. WGS was conducted at Centro Nacional de Análisis Genómico (CNAG, Barcelona, Spain) and stored at Navarra de Servicios y Tecnología (NASERTIC, Navarre, Spain).

### 2.3. Whole Exome Sequencing and Data Analysis

In the *pharmaNAGEN* project, germline DNA was extracted from saliva or blood samples using a DNA Blood Maxi Kit (Qiagen, Hilden, Germany) and sequenced with a Nextera DNA Exome kit. The raw data were aligned to the GRCh37/hg19 genome, sourced from UCSC (https://genome.ucsc.edu/, accessed on 4 September 2019), utilizing BWA [13]. The resulting BAM files were marked using Picard (https://broadinstitute.github.io/picard/, accessed on 4 September 2019). Utilizing GATK v4.1.0 [12], an updated version, eliminates the need for the indel realignment step. Recalibration and variant calling were executed with BQSR and Haplotype tools. This process yielded the final gVCF file for each sample. WES was conducted at CNAG and stored at NASERTIC.

### 2.4. Individual Selection

Selection criteria included unrelated individuals with Spanish ancestry. To assess relatedness among individuals, identity by descent (IBD) was calculated using the method of moments (MoM) with the R package SNPRelate [14]. For validation of ethnicity, the CSVS tool [9] was used to determine the degree of alignment of a sample with the genetic variability of the Spanish population. Individuals with a score equal to or higher than 0.9 were categorized as being of Spanish ancestry.

Applying these selection criteria to the *NAGEN1000* project resulted in a cohort of 358 participants denominated NAVARREsel. Within this group, 127 individuals had been diagnosed with various monogenic diseases, and the most prevalent conditions were polycystic kidney disease (14/127), breast cancer (10/127), and hereditary ataxia (8/127).

Regarding the validation dataset, named NAVARREval, the same inclusion criteria were applied to the *pharmaNAGEN* project, resulting in 239 participants with Crohn’s disease (153/239) and/or ulcerative colitis (86/239).

### 2.5. Variant Quality Control and Filtering in NAVARREsel

A 358 multi-sample gVCF was generated with the NAVARREsel cohort, and biallelic SNVs located in exonic regions were selected. The targeted exonic interval was extracted from Nextera (https://support.illumina.com/sequencing/sequencing_kits/nextera-dna-exome/downloads.html, downloaded on 27 September 2023). Variants with a read depth of less than 10, a genotype-quality score below 50, or a call rate of less than 100% were removed. Variants on the X and Y chromosomes were eliminated to avoid sex bias, as well as the mitochondrial chromosome, given its complexity. The Hardy–Weinberg equilibrium (HWE) score [15] was calculated using PLINK (-hardy), and SNVs significantly deviated with *p*-value < 10^−5^ were excluded.

### 2.6. Variant Quality Control and Filtering in NAVARREval

Genotype information for specific SNVs from NAVARREval samples was extracted using the SelectVariants tool of GATK v4.1.0 [12]. SNVs with a call rate lower than 80% and those exhibiting a significant deviation from HWE (*p*-value < 10^−5^) were excluded from validation.

### 2.7. Variant Annotation

Variants were annotated using ANNOVAR (version available on 24 October 2019, https://annovar.openbioinformatics.org/) [16]. The identification of known variants was performed using the dbSNP database (version GCF_000001405.25, downloaded from https://ftp.ncbi.nih.gov/snp/latest_release/VCF/ accessed on 30 September 2023) [17]. SNVs were also annotated: (1) for clinical significance by referring to ClinVar (v.20230930, https://www.ncbi.nlm.nih.gov/clinvar/, accessed on 2 October 2023) [18], OMIM (downloaded on 2 October 2023, https://www.omim.org/) [19], VarSome [20], and Franklin [21], and (2) for pharmacological relevance using PharmGKB (https://www.pharmgkb.org/, accessed on 2 October 2023) [22]. Additionally, the pathogenicity of the variants was evaluated using CADD (v1.6, https://cadd.gs.washington.edu/, accessed on 2 October 2023) [23], REVEL score (v1.3, https://zenodo.org/records/7072866, accessed on 2 October 2023) [24], spliceAI [25], and Polyphen2 (http://genetics.bwh.harvard.edu/pph2/dokuwiki/downloads downloaded on 4 October 2023) [26]. Variants associated with Crohn’s disease and/or ulcerative colitis were identified by referencing the Inflammatory Bowel Disease (IBD) database (accessed on 10 October 2023, from https://www.cbrc.kaust.edu.sa/ibd/index.php?p=ibd#) [27].

### 2.8. Population Projects

The MGP project was a Spanish initiative, primarily featuring WES data from 267 healthy and unrelated participants, mainly from Andalusia and Galicia (Spanish regions) [8].

The population data from 1KGP phase 3 encompassed 2504 genome samples representing 26 populations [28]. European populations within this dataset included 503 samples from Europe (EUR), such as British in England and Scotland (GBR), Finnish in Finland (FIN), Iberian population in Spain (IBS), Toscani in Italy (TSI), and Utah residents with Northern and Western European ancestry (CEU). Additionally, there were 504 samples from East Asia (EAS), 489 from South Asia (SAS), 661 from Africa (AFR), and 347 from America (AMR), covering various populations.

The gnomAD genomes project v2.1.1 comprises 15,691 genomes and represents diverse populations worldwide, including Africans, Americans, Asians, and Europeans. Within European populations, the majority originated from north-western Europe, Estonia, Finland, and a smaller representation from southern Europe [4].

### 2.9. Population Frequencies

Based on the population projects described in the previous section, three populations were generated for this study as reference: (1) gnomAD, with the original frequency from the gnomAD genome project; (2) *1KGP_noIBS*, with the mean of all 1KGP population’s frequencies, excluding the IBS cohort of 107 individuals; and (3) *spain*, combining the frequencies of the IBS and MGP cohorts. The integration process for the *spain* population consisted of adding the number of total alternate alleles divided by the sum of the total number of alleles across the two cohorts.

### 2.10. Principal Components Analysis, Admixture, and F_ST_ Analysis

The original VCF files for each chromosome from the 1KGP phase 3 were downloaded on 27 September 2023, from https://ftp.1000genomes.ebi.ac.uk/vol1/ftp/release/20130502/. Subsequently, these files were merged using the Picard MergeVCFs tool. Access to the raw data from MGP was granted upon request, and the multi-sample VCF was retrieved for the EGA repository at https://ega-archive.org/datasets/EGAD00001003101, accessed on 27 September 2023. Then, the VCF files from 1KGP, MGP, and NAVARREsel were combined using the GATK tool’s CombineGVCFs function. The resulting combined file was used to perform Principal Component Analysis (PCA) with R libraries SNPRelate and SNPassoc, conduct ADMIXTURE (v1.3.0) [29] from 3 to 7 genetic components (K), and calculate the mean pairwise F_ST_ values between populations using vcftools (v0.1.17) [30].

### 2.11. Enrichment Analysis

Functional enrichment, including pathway (KEGG), biological process (GO), disease (OMIM), and human phenotype ontology (HPO), was performed using WebGestalt (https://www.webgestalt.org/, accessed on 2 October 2023) [31].

## 3. Results

### 3.1. Navarrese Discovery Cohort

The *NAGEN1000* WGS Navarrese project was composed of 688 individuals from 294 families (mainly trios) with a rare disease. The WGS was conducted with a mean coverage of 30X, providing comprehensive genomic data across the entire genome. For our study, a subset of this cohort was selected satisfying two criteria: unrelatedness and Spanish ancestry. This result yielded 358 individuals, referred to as NAVARREsel.

Then, biallelic SNVs on chromosomes 1 to 22, from the exonic region covered by the Nextera Exome Enrichment kit, were extracted. Subsequently, variants with read depth < 10, genotype quality < 50, or missing genotype in at least one sample were filtered out. Additionally, sites significantly deviated from Hardy–Weinberg equilibrium (HWE, *p*-value < 10^−5^) were removed [32]. Finally, 61,410 SNVs remained, of which 21,174 were identified as common variants (MAF > 1%). We observed that including additional individuals did not reveal new common variants, and 21,174 were achieved when considering over 100 individuals (Figure S1a).

### 3.2. Genetic Variation between Navarre, Spanish, and Global Populations

**Figure 2:**
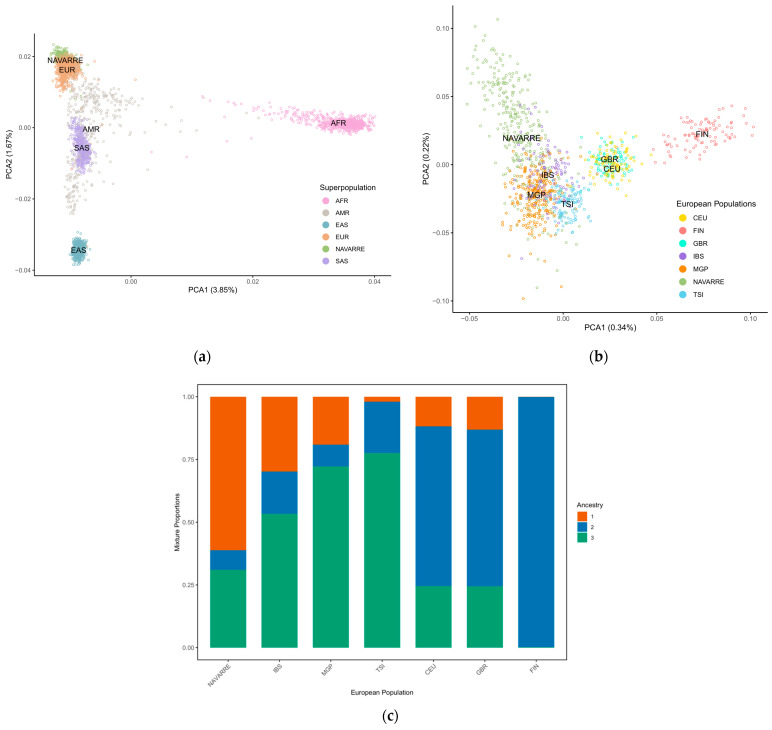
(**a**) Principal component analysis of overlapped variants between NAVARREsel, MGP, and 1KGP (including all populations), and coloured by superpopulations. (**b**) Principal component analysis of overlapped variants between NAVARREsel, MGP, and 1KGP (including exclusively European populations). (**c**) Genetic admixture analysis of 1128 individuals from 7 European populations for the optimal *K* value = 3. Abbreviations: PCA, principal component analysis; AFR, African populations; AMR, American populations; EAS, east-Asian populations; SAS, south-Asian populations; EUR, European populations; IBS, Iberian populations in Spain; MGP, Medical Genome Project; TSI, Toscani in Italy; CEU, Utah residents with Northern and Western European ancestry; GBR, British in England and Scotland; FIN, Finnish in Finland.

We performed a principal component analysis (PCA) on the shared variants between NAVARREsel, 1KGP, and MGP to depict its relationship. We observed a clear distinction between Navarre and Asian/African populations, reflecting established genetic differences (Figure 2a). Conversely, an overlap is observed between Navarre and European populations, emphasizing their genetic affinity. Thus, focusing on European populations (Figure 2b), we observed that Navarrese individuals are close to the Spanish populations (IBS and MGP) and exhibit proximity to Italian individuals (TSI). Supplementary Figure S2a,b illustrate plots PCA1 against PCA3, and PCA2 against PCA3. This observation is supported when estimating the ancestries of the European populations using ADMIXTURE [29]. The average number of ancestries in each population was calculated with the optimal component *K* = 3, which yielded the lowest cross-validation error (Figure 2c and Figure S3 admixture result at individual level). The Navarrese population showed the highest ancestral proportion on component 1 (61%). In the IBS and MGP populations, component 1 decreased to 30% and 20%, respectively, and was nearly absent (0.1%) in the FIN population. In contrast, component 2 was predominant in the FIN population (99%), while being the lowest in the Navarrese cohort (7%).

To further analyse the genetic differentiation, we calculated the mean pairwise F_ST_ values. The lower F_ST_ value indicates greater similarity between populations. This occurred when comparing Navarre with the Spanish (F_ST(Navarre-IBS)_ = 0.0001 and F_ST(Navarre-MGP)_ = 0.0007) and Italian (F_ST(Navarre-TSI)_ = 0.0014) populations. In contrast, the highest differentiation was observed against East Asian and African populations (F_ST(Navarre-EAS)_ = 0.0328, F_ST(Navarre-AFR)_ = 0.0434, Table S1).

These findings, aligning with biological expectations, underscore the regional and continental genetic affinities, providing insights into historical populations and evolutionary dynamics.

### 3.3. Exclusive Common Variants in Navarre

**Figure 3:**
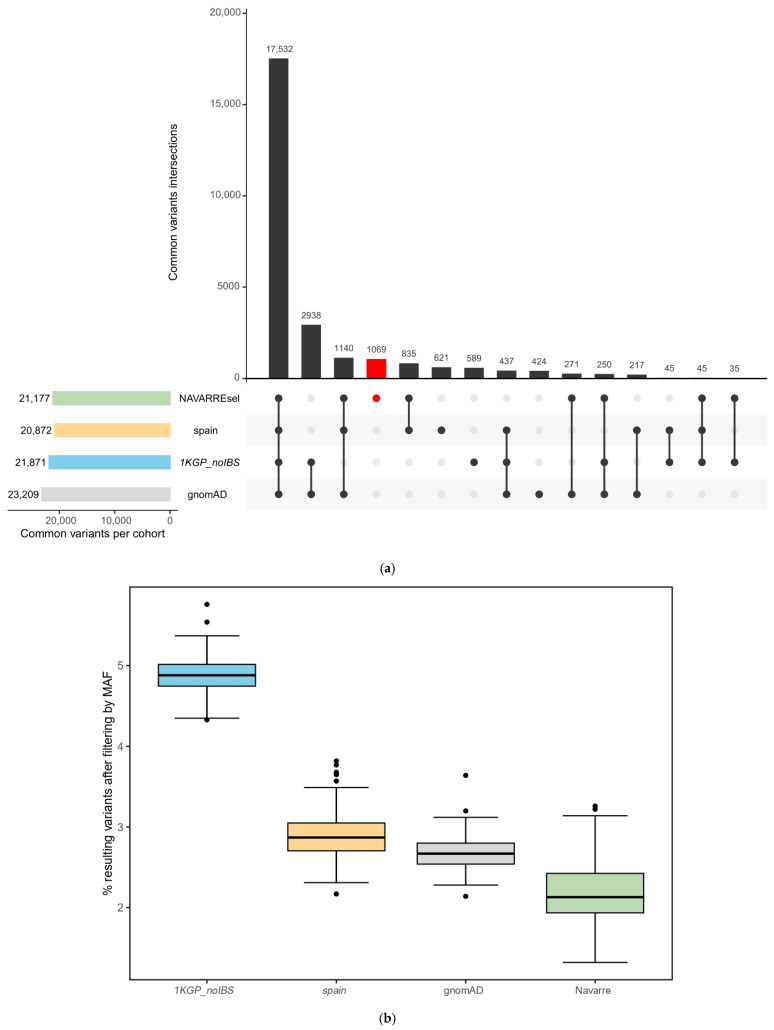
(**a**) Upset plot of common variants (MAF > 1%) of each population: NAVARREsel, *spain*, *1KGP_noIBS*, and gnomAD. (**b**) Resulting percentage of variants per patient (*n* = 127) after removing common variants from Navarre, *spain*, gnomAD, or *1KGP_noIBS* populations. The box plots represent the median, upper, and lower quartiles with the centre line and box bounds, respectively. Whiskers display the largest and smallest values within 1.5 times the interquartile range from the quartiles. Abbreviations: *1KGP_noIBS*, 1000 Genomes Project without Iberian population; gnomAD, Genome Aggregation Database; *spain*, integration of IBS and MGP populations.

To identify exclusive Navarrese common variants, we examined allele frequency among the Navarre population and the three referenced populations: *1KGP_noIBS*, gnomAD, and *spain*. A comparison of the MAF revealed that most variants (17,532 SNVs) were classified as common (MAF > 1%) across the four populations. However, 835 variants exhibited higher prevalence solely in Spanish cohorts (Navarre and *spain*). Specifically, 1069 SNVs were identified as common in Navarre, and rare (MAF ≤ 1%) in the rest (Figure 3a).

To validate these 1069 variants, we used the NAVARREval cohort, a subset of 239 WES samples from the *pharmaNAGEN* project. The validation cohort consists of unrelated individuals of Spanish descent from the current Navarrese population diagnosed with Crohn’s disease (159/239) or ulcerative colitis (86/239). Before validation, we assessed the association of these SNVs with these conditions by cross-referencing them with reported variants in the Inflammatory Bowel Disease database, which catalogues variants highly linked to the mentioned diseases [27]. The absence of the 1069 SNVs in this database ensured an unbiased and robust validation process.

Among the 1069 variants initially identified, 998 were detected in NAVARREval with a call rate greater than 80% and demonstrated conformity to HWE. Notably, 676/998 of these SNVs (68%; *p*-value = 2.2 × 10^−16^) were consistently classified as common in NAVARREval, confirming their prevalence within the Navarrese population (variants’ information in Table S2). The validation cohort was sufficient to validate the Navarrese common variants, reaching a plateau in Figure S1b. On the contrary, within the non-validated subset (322/998, 32%), 134 SNVs exhibited MAFs in NAVARREsel that did not exceed a 2-fold difference in NAVARREval, indicating close MAF between both datasets (Figure S4). This exploration of MAF patterns ensures a comprehensive understanding of the genetic landscape within the Navarre population and its stability across different datasets. The MAF spectrum for these sets is depicted in Supplementary Figure S5.

### 3.4. Characterization of Common Navarrese Variants

The annotation of the 676 common Navarrese SNVs revealed 227 synonymous, 371 missense, and 5 loss-of-function (LoF) variants. These LoF variants were not reported in the ClinVar database [18] and were located in five distinct genes without an associated phenotype, according to OMIM [19]. Following the ACMG guidelines for variant classification, four were classified as variants of uncertain significance (VUS) and one as benign [33].

Clinically, 264/676 SNVs were reported in ClinVar: 1/264 as a risk factor, 181/264 as benign/likely benign, 32/264 as VUS, 48/264 as having conflicting interpretations, and 2/264 as likely pathogenic. These likely pathogenic missense variants were in *SCNN1B* [c.1688G > A p.Arg563Gln; MAFNAVARREsel = 0.013, MAFNAVARREval = 0.016] and in *PTGIS* [c.824G > A p.Arg275Gln; MAFNAVARREsel = 0.013, MAFNAVARREval = 0.021], associated with “low renin hypertension” and “childhood-onset schizophrenia”, respectively, according to ClinVar [18]. The evidence supporting these associations is limited, with a score of 1 out of 4 as reviewed by a single submitter record. Therefore, given its notable prevalence in the Navarrese population, being observed in healthy and affected (not related to this phenotype) individuals, these variants might be reconsidered and reclassified as VUS under the ACMG guidelines [34].

*In silico* analysis of the 676 variants with functional predictors revealed eight variants as pathogenic by three different pathogenicity tools (REVEL_score > 0.8 [24], CADD > 20 [23], and Polyphen indicating “probably” or “possibly”) [26]. However, a comprehensive examination of clinical databases, including ClinVar [18], Varsome [20], and Franklin [21], contradicted these predictions based on ACMG criteria. Instead, the majority of these variants were classified as uncertain significance (1/8), likely benign (5/8), and benign (2/8). This suggests that these variants are not disease causing.

Additionally, the variant in *BPIFB3* [c.387-1G > T; MAFNAVARREsel = 0.01536, MAFNAVARREval = 0.02092], predicted to impact the canonical splicing acceptor site (spliceAI score = 0.99), was reclassified as benign based on the allele frequency and the number of homozygotes as per ACMG criteria, indicating no clinical relevance [25].

Moreover, common Navarrese variants showed no impact on drug metabolism/efficacy, according to PharmGKB [22]. They did not exhibit significant enrichment in pathways, biological processes, related diseases, or phenotypic ontologies.

### 3.5. Refining Disease-Causing Variant Identification in the Navarrese Population

We identified common variants in the Navarrese population, highlighting population-specific importance in advancing personalized medicine. The aim was to improve the identification of disease-causing variants during genetic diagnosis using NGS. Therefore, we selected 127 WGS Navarrese patients from the *NAGEN1000* project diagnosed with rare disorders and extracted exonic SNVs on chromosomes 1 to 22, averaging 8871 variants per patient.

We refined the variant list by excluding common variants (MAF > 1%) from *1KGP_noIBS*, gnomAD, *spain*, and Navarre. The Navarrese filtering emerged as the most stringent, resulting in 2.1% of the initial set, compared to 2.7% with gnomAD, 2.9% with *spain* frequencies, and the least restrictive, 4.9% with *1KGP_noIBS* (Figure 3b). This underscores the effectiveness of the Navarrese-specific filter in prioritizing and streamlining genetic investigations.

## 4. Conclusions

In this study, we aimed to enhance diagnostic precision in the current Navarrese population by exploring common population-specific variants. Utilizing WGS data from 358 individuals of Navarre, we identified 61,410 SNVs, with 21,174 being common. Genetic analysis shows affinity with European populations and low differentiation with Spanish populations.

Focusing on exclusively common variants in residents in Navarre compared with referenced populations, we obtained 1069 SNVs, of which 676 were validated in another Navarrese cohort. Of these, none showed clinical or pharmacological relevance beyond what was observed in the Spanish population [35]. This aligns with the expectation that common population variants are less likely to be associated with disease ethology.

Our findings underscore the relevance of considering population-specific factors in genomic diagnostics, which provides complementary insights alongside pangenome references [36]. However, the study would benefit from being expanded to include a larger cohort of participants (to provide greater statistical power to identify common variants) and increase the number of healthy individuals. In conclusion, by identifying and excluding common variants within the Navarrese population, we have successfully refined the identification of potential disease-causing variants, contributing to the advancement of personalized medicine for individuals from Navarre. Further research will enhance these insights for broader applications.

## Data Availability

Data is available from the corresponding author upon reasonable request.

## Supplementary Materials

The following supporting information can be downloaded at: https://www.mdpi.com/article/10.3390/genes15050585/s1, Figure S1: Accumulative number of new variants contributed by individuals. (a) All common variants in NAVARREsel (21,174 SNVs). (b) The 676 variants are exclusively common in NAVARREsel and validated in NAVARREval. Figure S2: (a) Principal component analysis of overlapped variants between NAVARREsel, MGP, and 1KGP (including all populations), and coloured by superpopulations. (b) Principal component analysis of overlapped variants between NAVARREsel, MGP, and 1KGP (including exclusively European populations). Figure S3: Genetic admixture analysis of 1128 individuals from 7 European populations, at an individual level, for the optimal *K* value = 3. Figure S4: Comparison of MAF between NAVARREsel and NAVARREval encompassing both validated and non-validated variants, with a total of 998 SNVs. Figure S5: Minor allele frequency spectrum for the following distinct sets: 21,174 common variants in NAVARREsel (blue colour); 1069 common variants in NAVARREsel but not in *1KGP_noIBS*, gnomAD and spain (pink); and 676 common variants in both NAVARREsel (green) and NAVARREval (yellow). Common variants are defined with a MAF > 1%. Table S1: F_st_ values between Navarre against MGP and 1KGP populations. Table S2: Variants’ information of the 676 exclusively common SNVs of the Navarre population. Details: ”CHR”: chromosome; “Position”: variant position; “REF”: reference allele; “ALT”: alternate allele; “NAVARREsel_maf”: MAF in NAVARREsel cohort; “NAVARREval_maf”: MAF in NAVARREval cohort; “1KGP_phase3noIBS”: MAF in 1KGP excluding IBS cohort; “Spain”: MAF in spain cohort (combining IBS and MGP); ”gnomAD”: MAF in gnomAD; “Gene”: gene where the variant is located; “Type”: type of variant; “ExonicInfo”: exonic variant type, if applicable; “ClinVar”: ClinVar information; “SpliceAI”: Splice AI results; “REVEL”: REVEL results; “CADD”: CADD results; “Polyphen2”: Polyphen2 results.

## Appendix A

**Table A1:** NAGEN-Scheme.

Name	Department	Project
Anda Apiñaniz, Emma	Servicio de Endocrinología y Nutrición, HUN	NAGEN1000
Artigas López, Mercedes	Servicio de Genética Médica, HUN	NAGEN1000
Bandrés Elizalde, Eva	Servicio de Hematología, HUN	NAGEN1000
Basurte Elorz, M^a^ Teresa	Servicio de Cardiología, CHN	NAGEN1000
Brennan, Paul	NENC NHS Genomic Medicine Centre. Newcastle upon Tyne, UK.	NAGEN1000
Celaya Lecea, Concepción	Subdirección de Farmacia, SNS-O	pharmaNAGEN
Cuesta Zorita, Manuel Jesús	Servicio de Psiquiatría, Salud mental	NAGEN1000
Curi Chercoles, Sergio Miguel	Servicio de Neumología, HUN	NAGEN1000
De la Cruz Sánchez, Susana	Servicio de Oncología Médica, HUN	NAGEN1000
Erviti López, Juan	Subdirección de Farmacia, SNS-O	pharmaNAGEN
Fanlo Mateo, Patricia	Servicio de Medicina Interna, HUN	NAGEN1000
González, Luis Angel	AVANTIA 400+.	NAGEN1000
Gonzalo Etayo	Navarra de Servicios y Tecnología NASERTIC. Spain	NAGEN1000
Gorricho Mendívil, Javier	Subdirección de Farmacia, SNS-O	pharmaNAGEN
Guerra Lacunza, Ana	Servicio de Aparato Digestivo, HUN	NAGEN1000
Gut, Ivo	Centro Nacional de Análisis Genómicos CNAG. Spain	NAGEN1000
Ibáñez Bosch, Rosario	Servicio de Endocrinología y Nutrición, HUN	NAGEN1000
Jiménez, Jorge	Navarra de Servicios y Tecnología NASERTIC. Spain	NAGEN1000
Lasheras, Gorka	Navarra de Servicios y Tecnología NASERTIC. Spain	NAGEN1000
Lorea Bueno	Pharmamodelling	pharmaNAGEN
Maite Sarobe Carricas	Servicio de Farmacia Hospitalaria, HUN	pharmaNAGEN
Mendioroz Iriarte, Maite	Servicio de Neurología, HUN	NAGEN1000
Molinuevo Ruiz de Zarate, José Ignacio	Servicio de Oftalmología, HUN	NAGEN1000
Montes Díaz, Marta	Servicio de Anatomía Patológica, HUN	NAGEN1000
Navarro, Adela	Servicio de Cardiología, HUN	pharmaNAGEN
Onintza Sayar	Pharmamodelling	pharmaNAGEN
Pinillos, Iñaki	Navarra de Servicios y Tecnología NASERTIC. Spain	NAGEN1000
Purroy Irurzon, Carolina Eugenia	Servicio de Nefrología, HUN	NAGEN1000
Sagaseta de Ilurdoz Uranga, M^a^ Josefa	Servicio de Pediatría, HUN	NAGEN1000
Santesteban Muruzabal, Raquel	Servicio de Dermatología, HUN	NAGEN1000
Vicuña, Miren	Servicio de Digestivo, HUN	pharmaNAGEN
Viguria, M^a^ Cruz	Servicio de Hematología, HUN	pharmaNAGEN
Yoldi Petri, M^a^ Eugenia	Servicio de Pediatría, HUN	NAGEN1000
Zubicaray Ugarteche, José Jacinto	Servicio de Otorrinolaringología, HUN	NAGEN1000
Zudaire, Maite	Servicio de Hematología, HUN	pharmaNAGEN
