# Supplementary material for: Characterization of the Common Genetic Variation in the Spanish Population of Navarre
Source: Genes (Basel). 2024 May 4;15(5):585. doi: 10.3390/genes15050585 (PMC11121068; doi:10.3390/genes15050585)
Supplement: Supplementary file 1 [file genes-15-00585-s001.zip › SupplementaryFiles/NavarreVariantsSupInf.pdf]

Characterization of the common genetic variation among the Spanish population of Navarre

Supplementary Information

Supplementary Figures

Figure S1: Accumulative number of new variants contributed by individuals **a)** All common variants in NAVARREsel (21,174 SNVs). **b)** The 676 variants are exclusively common in NAVARREsel and validated in NAVARREval.

Figure S2: **a)** Principal component analysis of overlapped variants between NAVARREsel, MGP, and 1KGP (including all populations), and coloured by superpopulations. **b)** Principal component analysis of overlapped variants between NAVARREsel, MGP, and 1KGP (including exclusively European populations).

Figure S3: Genetic admixture analysis of 1128 individuals from 7 European populations, at an individual level, for the optimal *K* value = 3.

Figure S4: Comparison of MAF between NAVARREsel and NAVARREval encompassing both validated and non-validated variants, with a total of 998 SNVs.

Figure S5: Minor allele frequency spectrum for the following distinct sets: 21,174 common variants in NAVARREsel (blue colour); 1069 common variants in NAVARREsel but not in *1KGP_noIBS*, gnomAD and *spain* (pink); and 676 common variants in both NAVARREsel (green) and NAVARREval (yellow). Common variants are defined with a MAF > 1%.

Supplementary Tables

Table S1: F_{st} values between Navarre against MGP and 1KGP populations.

Table S2: Variants' information of the 676 exclusively common SNVs of the Navarre population. Details: "CHR": chromosome; "Position": variant position; "REF": reference allele; "ALT": alternate allele; "NAVARREsel_maf": MAF in NAVARREsel cohort; "NAVARREval_maf": MAF in NAVARREval cohort; "1KGP_phase3noIBS": MAF in 1KGP excluding IBS cohort; "Spain": MAF in *spain* cohort (combining IBS and MGP); "gnomAD": MAF in gnomAD; "Gene": gene where the variant is located; "Type": type of variant; "ExonicInfo": exonic variant type, if applicable; "ClinVar": ClinVar information; "SpliceAI": Splice AI results; "REVEL": REVEL results; "CADD": CADD results; "Polyphen2": Polyphen2 results.

Figure S1

a

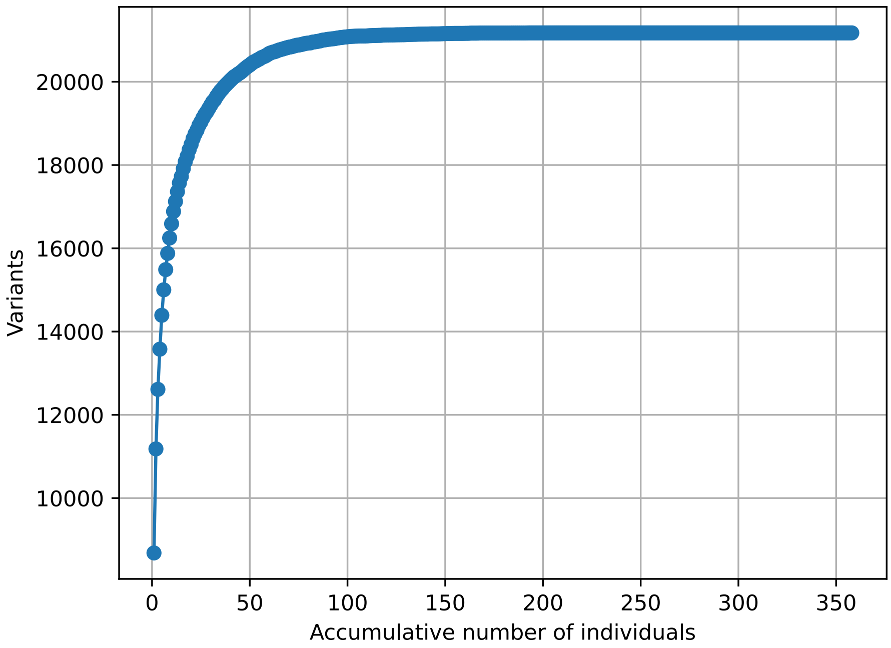

20000
18000
16000
14000
12000
10000
Variants
0
50
100
150
200
250
300
350
Accumulative number of individuals

b

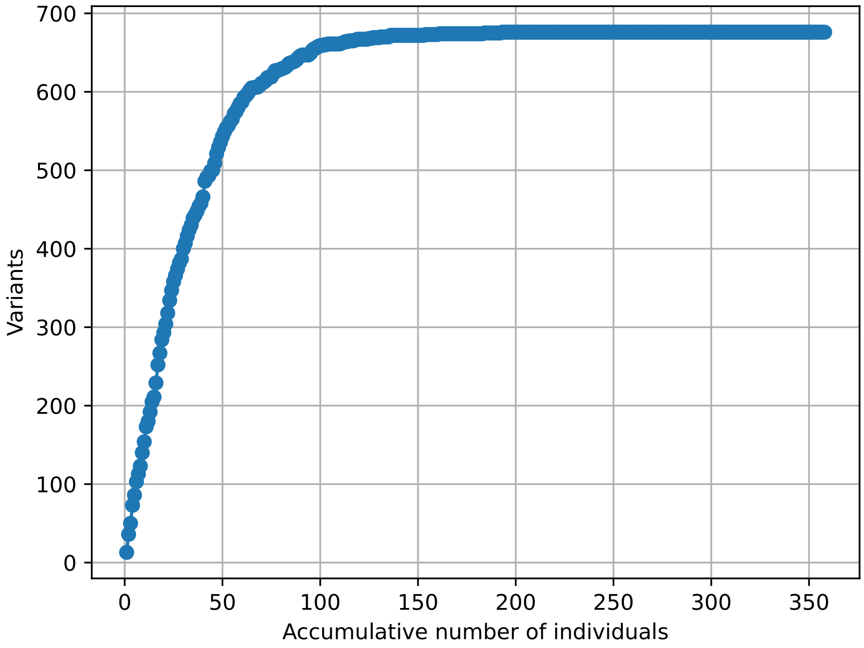

700
600
500
400
300
200
100
0
Variants
0
50
100
150
200
250
300
350
Accumulative number of individuals

Figure S2

a

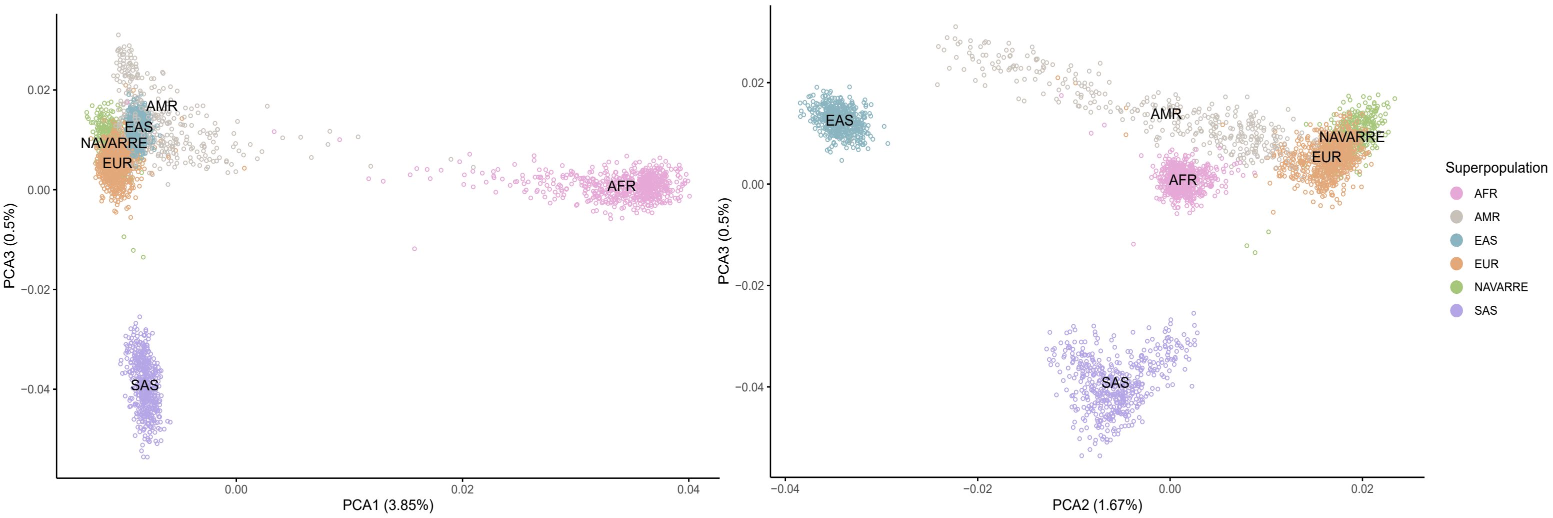
AMR
EAS
NAVARRE
EUR
AFR
SAS
PCA3 (0.5%)
PCA1 (3.85%)
PCA2 (1.67%)
Superpopulation
AFR
AMR
EAS
EUR
NAVARRE
SAS

b

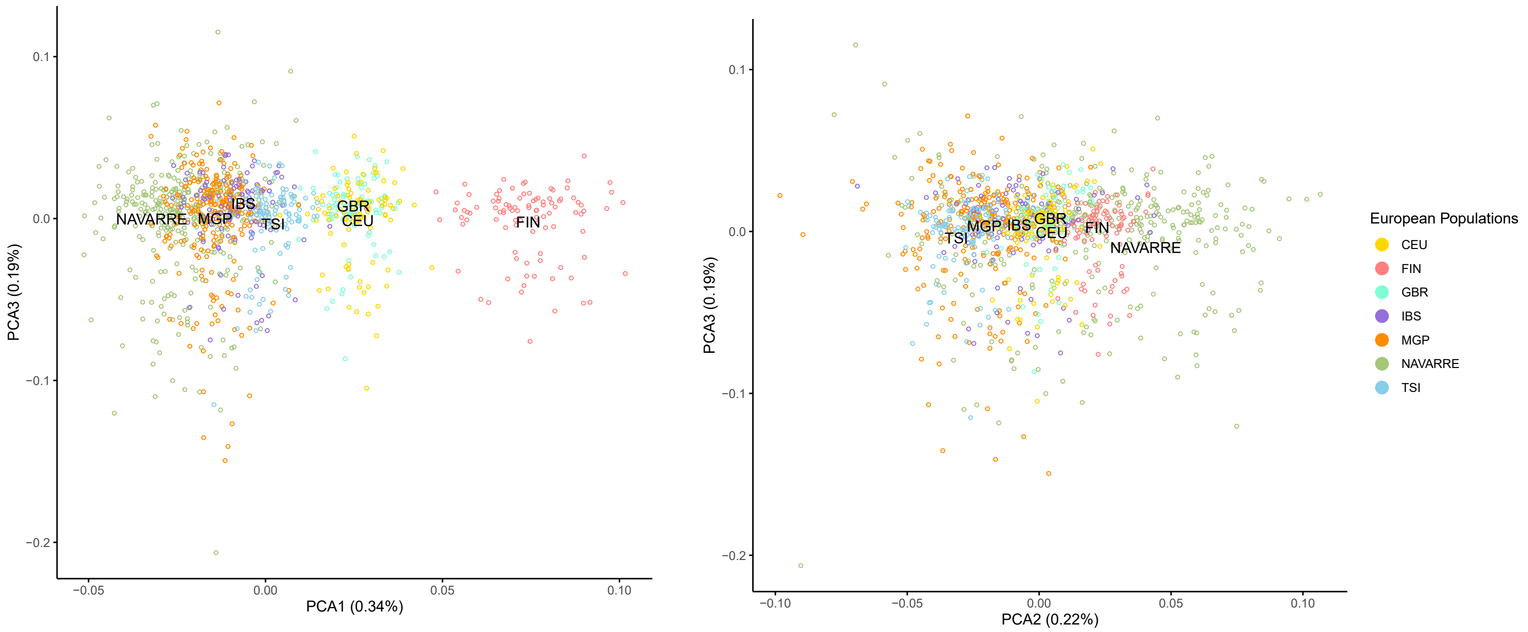
NAVARRE
MGP
IBS
TSI
GBR
CEU
FIN
PCA3 (0.19%)
PCA1 (0.34%)
PCA2 (0.22%)
European Populations
CEU
FIN
GBR
IBS
MGP
NAVARRE
TSI

Figure S3

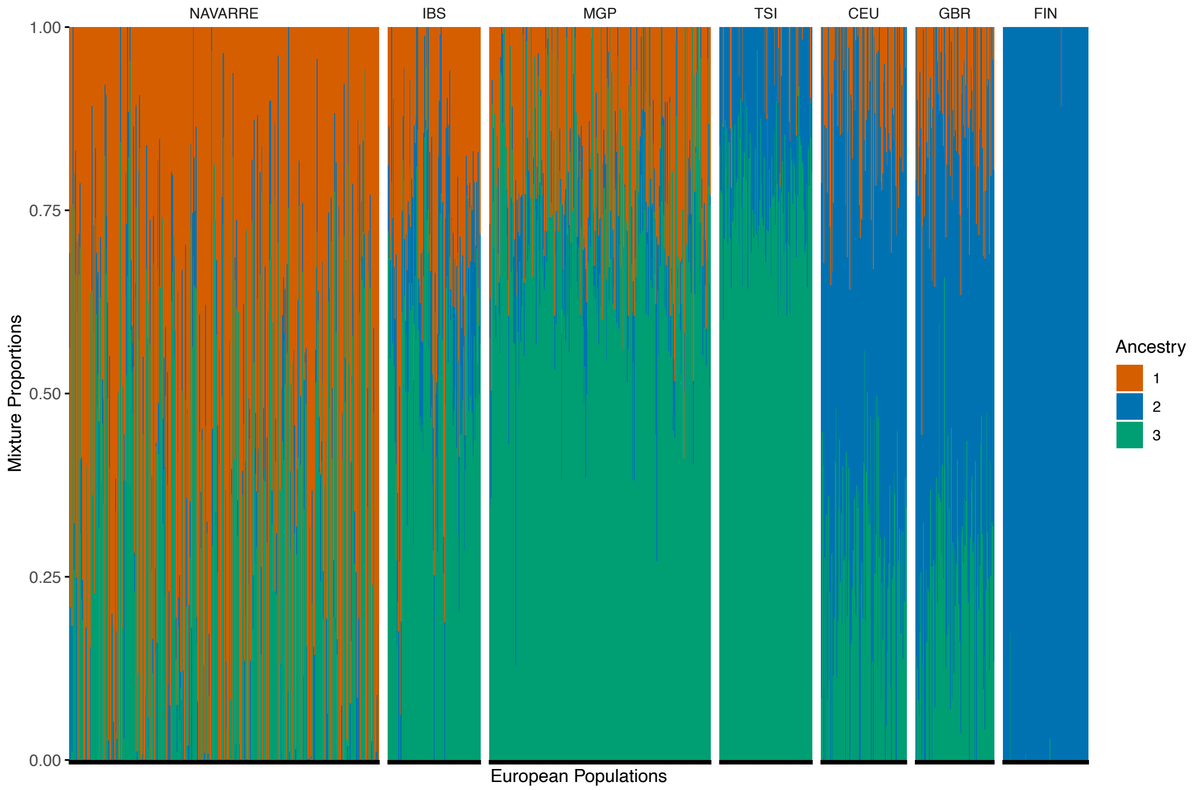
NAVARRE
IBS
MGP
TSI
CEU
GBR
FIN
1.00
0.75
0.50
0.25
0.00
Mixture Proportions
European Populations
Ancestry
1
2
3

Figure S4

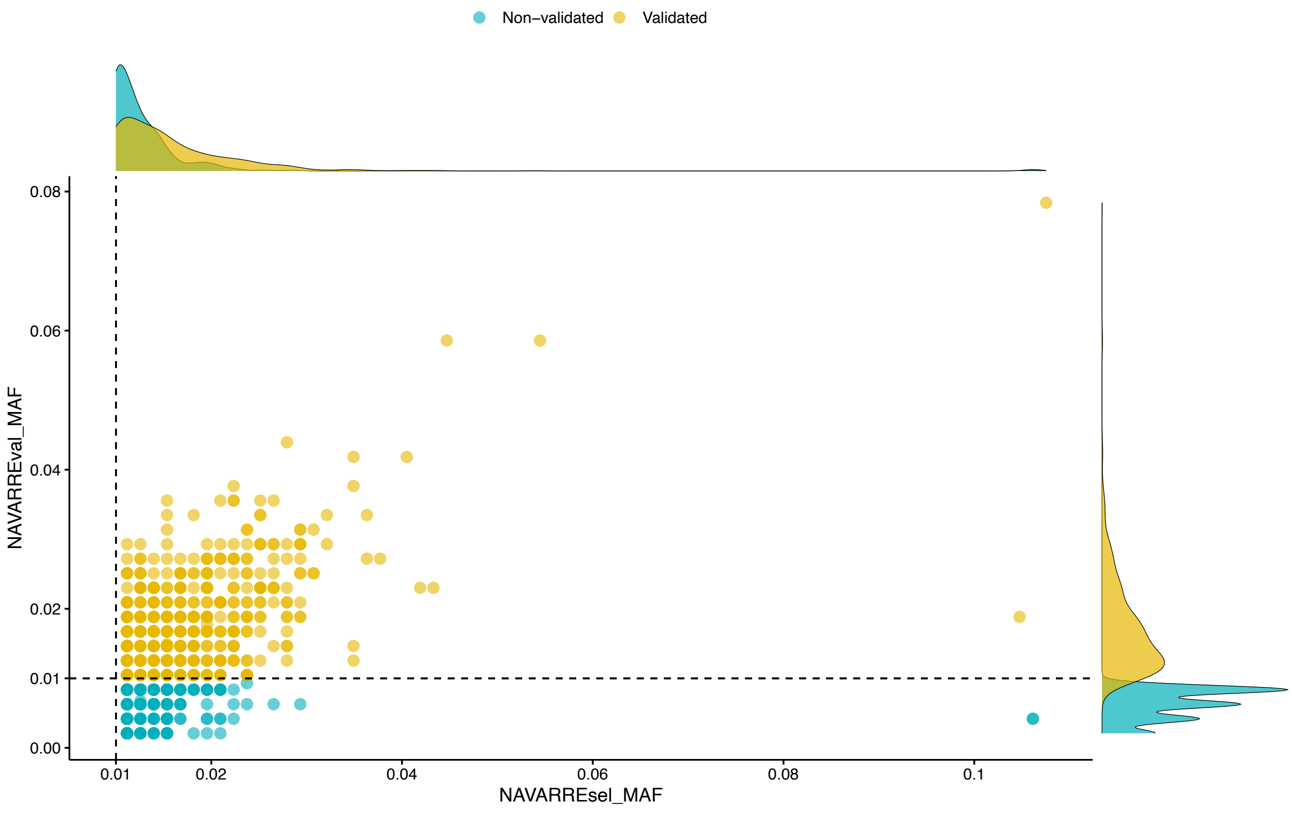
Non−validated
Validated
0.08
0.06
0.04
0.02
0.01
0.00
NAVARREval_MAF
0.01
0.02
0.04
0.06
0.08
0.1
NAVARREsel_MAF

Figure S5

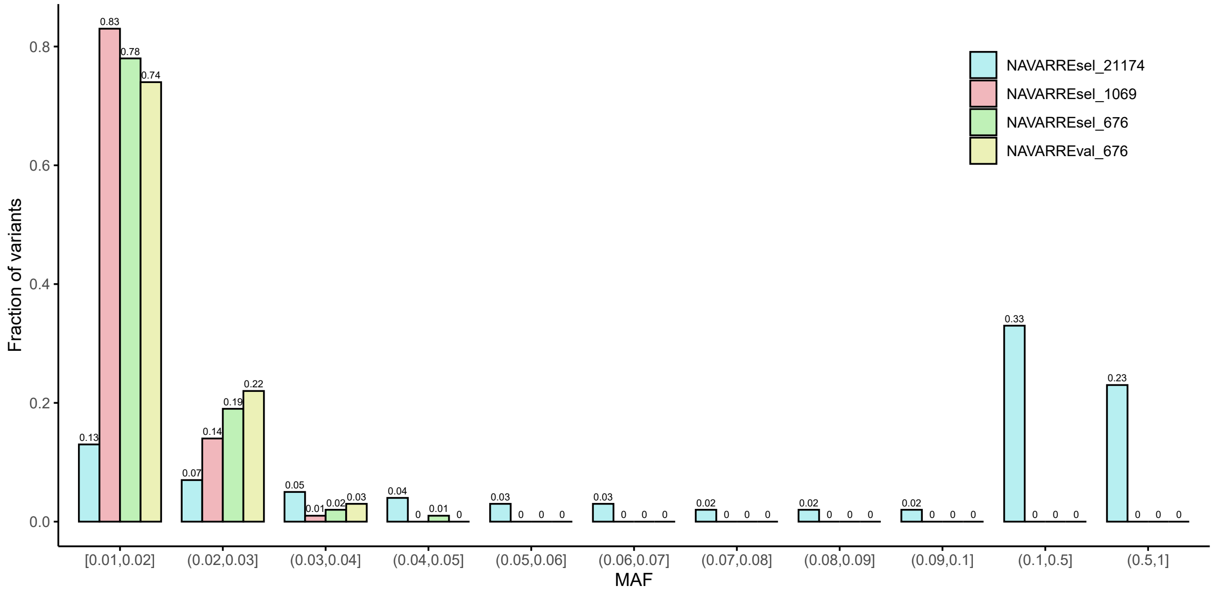

Fraction of variants
MAF
NAVARREsel_21174
NAVARREsel_1069
NAVARREsel_676
NAVARREval_676
0.0
0.2
0.4
0.6
0.8
[0.01,0.02]
(0.02,0.03]
(0.03,0.04]
(0.04,0.05]
(0.05,0.06]
(0.06,0.07]
(0.07,0.08]
(0.08,0.09]
(0.09,0.1]
(0.1,0.5]
(0.5,1]
